# Assessment of Coronary Inflammation by Pericoronary Fat Attenuation Index in Clinically Suspected Myocarditis with Infarct-Like Presentation

**DOI:** 10.3390/jcm10184200

**Published:** 2021-09-16

**Authors:** Anna Baritussio, Francesco Vacirca, Honoria Ocagli, Francesco Tona, Valeria Pergola, Raffaella Motta, Renzo Marcolongo, Giulia Lorenzoni, Dario Gregori, Sabino Iliceto, Alida L. P. Caforio

**Affiliations:** 1 Department of Cardiac, Thoracic, Vascular Sciences and Public Health, University of Padua, 35121 Padua, Italy; anna.baritussio@aopd.veneto.it (A.B.); francesco.vacirca@studenti.unipd.it (F.V.); francesco.tona@unipd.it (F.T.); valeria.pergola@aopd.veneto.it (V.P.); sabino.iliceto@unipd.it (S.I.); 2 Unit of Biostatistics, Epidemiology and Public Health, Department of Cardiac, Thoracic, Vascular Sciences and Public Health, University of Padua, 35121 Padua, Italy; honoria.ocagli@unipd.it (H.O.); giulia.lorenzoni@unipd.it (G.L.); dario.gregori@unipd.it (D.G.); 3 Department of Medicine (DIMED), University of Padua, 35121 Padua, Italy; raffaella.motta@unipd.it; 4 Haematology and Clinical Immunology, Department of Medicine, University of Padua, 35121 Padua, Italy; renzo.marcolongo@aopd.veneto.it

**Keywords:** coronary inflammation, myocarditis, coronary computed tomography angiography

## Abstract

Background: The pathophysiology of angina-like symptoms in myocarditis is still unclear. Perivascular fat attenuation index (pFAI) by coronary computed tomography angiography (CCTA) is a non-invasive marker of coronary inflammation (CI) in atherosclerosis. We explored the presence of CI in clinically suspected myocarditis with infarct-like presentation. Methods: We retrospectively included 15 consecutive patients (67% male, age 30 ± 10 years) with clinically suspected infarct-like myocarditis who underwent CCTA to rule out coronary artery disease. Right coronary artery (RCA) pFAI mean value was compared with that of healthy volunteers. Results: Mean RCA pFAI value was −92.8 ± 8.4 HU, similar to that of healthy volunteers (−95.2 ± 6.0, *p* = 0.8). We found no correlation between RCA pFAI mean values and peak Troponin I (r = −0.43, *p* = 0.11) and C-reactive protein at diagnosis (r = −0.25, *p* = 0.42). Patients with higher pFAI values showed higher biventricular end-systolic volumes (ESV) (*p* = 0.038 for left and *p* = 0.024 for right ventricle) and lower right ventricular ejection fraction (RVEF) (*p* = 0.038) on cardiovascular magnetic resonance. Conclusions: In clinically suspected myocarditis with infarct-like presentation, RCA pFAI values are lower than those validated in atherosclerosis. The correlation between higher pFAI values, higher biventricular ESV and lower RVEF, may suggest a role of pFAI in predicting non-atherosclerotic CI (i.e., infective/immune-mediated “endothelialitis”).

## 1. Introduction

Myocarditis is an inflammatory disease of the myocardium, with different aetiology, clinical presentation, and outcome [1]. Infarct-like presentation is common and is characterised by acute chest pain and electrocardiographic changes (ST/T wave changes) that can be accompanied by biventricular dysfunction and/or increased Troponin levels. The pathophysiology of angina-like symptoms in myocarditis with infarct-like presentation is, however, still unclear [1,2,3]. The pericoronary fat attenuation index (pFAI), as assessed by coronary computed tomography angiography (CCTA), has recently emerged as a non-invasive marker of coronary inflammation in coronary atherosclerosis, even at an early stage [4,5,6,7,8]. Pericoronary and epicardial adipose tissue are metabolically active tissues that can secrete inflammatory mediators, which determine a shift in tissue composition from a lipid towards an aqueous phase that is responsible for the tissue attenuation detected by CCTA [5,6,7,8,9,10]. The mechanisms of coronary inflammation, still poorly explored, may act both at the microcirculatory and epicardial artery levels. By analysing pFAI values by CCTA, we sought to explore the presence of coronary inflammation in clinically suspected myocarditis with infarct-like presentation, and to investigate possible correlations between pFAI and clinical and diagnostic features at diagnosis.

## 2. Material and Methods

### 2.1. Study Design and Participants

We retrospectively analysed our registry of 836 patients with clinically suspected (*n* = 465) and biopsy-proven myocarditis (*n* = 371), regularly followed-up, since 1993, at the Cardio-Immunology Outpatient Clinic of the University Hospital of Padua, Italy. We included in the analysis consecutive patients with clinically suspected myocarditis and infarct-like presentation who underwent CCTA at diagnosis, instead of coronary angiogram, to rule out coronary artery disease (CAD). Clinically suspected myocarditis was defined according to the 2013 European Society of Cardiology’s (ESC) position statement on myocarditis as follows: infarct-like presentation clinically suggestive of myocarditis and presence of at least one diagnostic criterion from different categories (electrocardiogram, ECG, echocardiogram, cardiovascular magnetic resonance) in the absence of angiographically detectable coronary artery disease (CAD) and known pre-existing cardiovascular disease or extra-cardiac causes that could explain the syndrome [1].

Infarct-like presentation was defined according to the 2013 ESC’s position statement on myocardial and pericardial diseases as a combination of the following features: (a) acute chest pain (in the absence of CAD); (b) ST/T wave changes on ECG (that, by definition, can be transient); (c) with/without normal global or regional LV and/or RV dysfunction on echocardiography or cardiovascular magnetic resonance; (d) with/without increased Troponin (Tn)T/TnI that may have a time course similar to acute myocardial infarction or a prolonged sustained release over several weeks or months [1].

Clinical, diagnostic, and imaging characteristics at diagnosis were collected. The study has been approved by the local Ethical Committee (Comitato Etico per la Sperimentazione Clinica della Provincia di Padova, protocol number 0070725, 24/11/2020) and has been performed in accordance with the Declaration of Helsinki.

### 2.2. Coronary Computed Tomography Angiography

CCTA was performed using a whole-heart scanner (Aquilion One Toshiba, Canon Medical Systems, Otawara, Japan). Computed tomography protocol scanning with prospective electrocardiography (ECG)-gating was performed during a breath-hold using 320 slices with a collimated slice thickness of 0.5 mm, using the standard protocol for coronary arteries. About 50–70 mL of non-ionic contrast agent (Iomeprolo 816.50 mg/mL, Iomeron 400, Bracco Imaging, Milan, Italy) was given to each patient. When the heart rhythm was higher than 65 bpm, metoprolol succinate (5–40 mg) was administrated intravenously unless contraindicated; all patients were also treated with sublingual nitroglycerin 5 min before the CT examinations.

Images were acquired after iodinated contrast administration using “bolus tracking” technique and reconstructed in post-processing by volume rendering technique (VRT) and curved multiplanar reformations with vessel centerline analysis.

**Figure 1:**
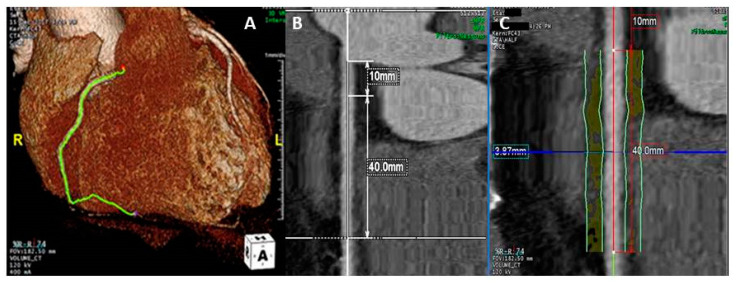
Pericoronary fat attenuation index (pFAI) measurement. The right coronary artery (RCA) is identified on the 3D reconstruction (**A**). The proximal 40 mm segment of the RCA is traced, starting 10 mm from the ostium (**B**). Perivascular fat is defined as the adipose tissue within a radial distance from the outer vessel wall equal to the diameter of the vessel (**C**). Perivascular FAI is then ascertained by quantifying the weighted perivascular fat attenuation based on the attenuation histogram of perivascular fat within the range −190 HU to −30 HU (not shown).

Pericoronary fat attenuation index (pFAI) was assessed using Aquarius Workstation version 4.4.13.P4 (TeraRecon Inc., Foster City, CA, USA). To measure pFAI we traced the proximal 40-mm segments of the three major epicardial coronary vessels, starting 10 mm from the ostium for right coronary artery (RCA); as previously reported, perivascular fat was defined as the adipose tissue within a radial distance from the outer vessel wall equal to the diameter of the vessel (Figure 1).

Perivascular FAI was ascertained by quantifying the weighted perivascular fat attenuation based on the attenuation histogram of perivascular fat within the range −190 HU to −30 HU, as previously described [4,5,6,7,8]. We measured pFAI in all three major coronary arteries, but restricted the analysis to the RCA as it has been proven to be representative of global coronary inflammation [11]. Two observers (A.B. and F.V.) measured pFAI in all three major coronary vessels, to assess inter-observer agreement.

Mean RCA pFAI values were compared to that of a healthy control group.

### 2.3. Statistical Analysis

Continuous variables were expressed as mean ± SD or median (IQR), as appropriate; categorical variables were expressed as *n* (%). Continuous variables were compared using Wilcoxon test, categorical variables were compared using chi-square test or Pearson test, for non-parametric variables. A *p*-value < 0.05 was considered statistically significant. The correlation between continuous variables was assessed with Pearson correlation test. Intraclass correlation coefficient (ICC) was used to test agreement between two observers on repeated pFAI measurements. *p*-value at 0.05 and 95% confidence interval were assessed both for Pearson correlation and ICC. All analysis was performed using the R software for statistical computing (R Foundation for Statistical Computing, Vienna, Austria, version 4.0.2) [12]. ICC was calculated using “irr” R package (R Studio, Boston, MA, USA, version 0.84.1) [13].

## 3. Results

### 3.1. Patients’ Characteristics

We included in the analysis 15 patients (67% male, mean age 30 ± 10 years) with clinically suspected myocarditis and infarct-like presentation who underwent CCTA to rule out the presence of CAD, which was excluded in all. At the time of diagnosis, nine patients complained of chest pain, three patients complained of syncope and two of palpitations; three patients had NYHA class II-IV. One patient had infero-lateral ST segment elevation and two patients showed inverted T waves in the lateral leads on ECG at presentation. All patients had abnormal high-sensitivity Troponin I (hs-TnI) at diagnosis (median 5197 ng/L (IQR 364–8469 ng/L), 10 patients had abnormal C reactive protein (CRP) levels (median 16.6 mg/dL (IQR 6.1–40.0 mg/dL). Anti-heart antibodies (AHA), tested in 10 patients (as per the 2013 ESC consensus [1]), showed weakly positive organ-specific AHA in four. The mean left ventricular (LV) ejection fraction (LVEF) on echocardiogram was normal (56.4 ± 9.5%), as was the right ventricular function (fractional area change, FAC, 46.2 ± 7.8%). The mean LVEF on cardiovascular magnetic resonance (CMR) was normal (57 ± 4%), as was the right ventricular ejection fraction (RVEF, 56 ± 4.7%); nine patients (64%) were found to have oedema on CMR, late gadolinium enhancement (LGE) was found in 12 patients (86%) with a median LGE mass of 6.5 g (IQR 2.5–7.9 g). Patients’ characteristics are summarised in Table 1.

To explore whether our study cohort was representative of our entire registry population, we compared it to the 450 clinically suspected myocarditis patients from our registry that did not undergo CCTA (instead receiving coronary angiography that ruled out CAD in all). Our cohort of patients undergoing CCTA was less likely to have had a viral infection in the 6 months preceding diagnosis (21% vs. 51%, *p* = 0.032) and more frequently had NYHA class II-IV at presentation (20% vs. 6%, *p* = 0.031). There was no difference in peak hs-TnI (5197 ng/L (IQR 364–8469) vs. 331 ng/L (IQR 88–1181), *p* = 0.12) and CRP levels at presentation (16.4 mg/dL (IQR 6.1–40.0) vs. 25.6 mg/dL (7.5–63.8), *p* = 0.27). There was no difference in biventricular volumes, as assessed by echocardiogram and CMR, while RVEF on CMR was lower in patients undergoing CCTA (56% vs. 60% in patients not undergoing CCTA, *p* = 0.048). Clinical and diagnostic characteristics of patients with clinically suspected myocarditis undergoing CCTA and of those not undergoing CCTA are summarised in Table 1.

### 3.2. Analysis of Pericoronary Adipose Tissue

We measured pFAI of the RCA in all patients, while pFAI around left anterior descending (LAD) and around left circumflex (LCx) could be measured in 14 and 7 patients, respectively. RCA mean pFAI value was −92.8 ± 8.4 Hounsfield Unit (HU), LAD mean pFAI value was −90.3 ± 6.1 HU, LCx mean pFAI value was −86.1 ± 9.4 HU (Table 2).

**Figure 2:**
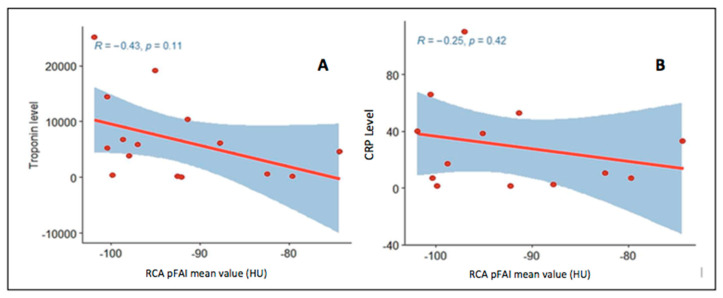
Correlation between RCA pericoronary fat attenuation index (pFAI) and peak Troponin I (**A** panel) and C reactive protein (CRP) levels (**B** panel) at diagnosis.

There was no difference in RCA mean pFAI value between our patients’ cohort and a control group of 13 healthy volunteers (85% male, mean age 41.2 ± 13.5 years): −92.8 ± 8.4 HU vs. −95.2 ± 6.0 HU, *p* = 0.80. We found no correlation between mean pFAI value and vessels diameter: RCA mean pFAI value and RCA diameter, R = −0.024 (95% CI −0.53–0.494), *p* = 0.932; LAD mean pFAI value and LAD diameter, R = 0.255 (95% CI −0.319–0.692), *p* = 0.379; LCx mean pFAI value and LCx diameter, R = 0.385 (95% CI −0.518–0.882), *p* = 0.394. There was no correlation between RCA mean pFAI values and peak hs-TnI (R = −0.43, 95% CI −0.77–0.111, *p* = 0.11) and CRP at diagnosis (R = −0.25, 95% CI −0.702–0.352, *p* = 0.42) (Figure 2).

Intraclass correlation coefficient was excellent for the measurement of pFAI mean values in all three coronary arteries (RCA ICC = 0.994, 95% CI 0.981–0.998; LAD ICC = 0.945, 95% CI 0.839–0.982; LCx ICC = 0.958, 95% CI 0.780–0.993) (Table 2), and it was good for the measurement of the diameters of all three coronary arteries (RCA diameter ICC = 0.851, 95% CI 0.623–0.947; LAD diameter ICC = 0.851, 95% CI 0.623–0.947; LCx diameter ICC = 0.825, 95% CI 0.253–0.968) (Table 2). Based on RCA median pFAI value [14] (−95.1 HU) we identified two groups: Group 1, patients with pFAI values inferior to RCA median pFAI value (*n* = 7), Group 2, patients with pFAI values greater or equal to RCA median pFAI value (*n* = 8) (Table 3).

There was no difference in demographic and clinical characteristics between the two groups, and no difference in clinical presentation. There was also no difference in peak hs-TnI (Group 1 5884 ng/L (IQR 4438–10,554) vs. Group 2, 2442 ng/L (IQR 146–7078), *p* = 0.26) and CRP levels (Group 1, 28.2 mg/dL (IQR 8.7–59.0) vs. Group 2, 10 mg/dL (IQR 4.0–35.2), *p* = 0.5). We found no difference in ECG and echocardiographic findings between the two groups. Based on CMR findings, patients with higher pFAI values (Group 2) showed greater biventricular end-systolic volumes (ESV) (LVESV 39.6 ± 7.3 mL/m^2^ vs. 33.5 ± 4.0 mL/m^2^, *p* = 0.024; RVESV 43.9 ± 8.2 mL/m^2^ vs. 32.8 ± 3.2 mL/m^2^, *p* = 0.024) and lower RVEF (53.6 ± 4.4% vs. 58.8 ± 3.3%, *p* = 0.038).

## 4. Discussion

In our cohort of patients with clinically suspected myocarditis and infarct-like presentation, we found mean RCA pFAI values which were lower than those recently reported in coronary atherosclerosis [11,14,15] and did not differ from those of a control group of healthy subjects (*p* = 0.80). To date, there are no data on normal pFAI values, although Ma et al. recently published pFAI values in 192 subjects with cardiovascular risk factors but without coronary plaques on CCTA [16]. In our study, intraclass correlation was excellent for pFAI values in all three major coronary arteries, in keeping with data from the literature that show excellent intra- and inter-observer agreement on repeated measurements [16,17]. There was no correlation between RCA mean pFAI values and peak Troponin I and CRP levels at diagnosis; this is in keeping with previous data from literature that showed no correlation between coronary inflammation, Troponin levels and systemic inflammation [11,15,18].

Pericoronary fat attenuation index has recently emerged as a novel non-invasive marker of coronary inflammation and is strongly associated with coronary atherosclerosis: pFAI values are higher around mixed coronary plaques and around culprit lesions in acute coronary syndromes [5,8,19]. Analysis of pFAI has shown to be a powerful diagnostic tool, including in asymptomatic patients, and a promising prognostic tool to assess patients’ cardiovascular risk [5,6,7,8,11,15].

We have recently reported lower pFAI values among patients with myocarditis, as compared to patients with myocardial infarction with non-obstructed coronaries (MINOCA) and Tako-Tsubo syndrome [20]. In the present cohort, RCA mean pFAI values are similar to those provided by Ma et al. in patients without coronary plaques on CCTA and to a cohort of healthy controls [16]. Based on pFAI values validated in coronary atherosclerosis [5,6,7,8,11,15,17], we may conclude that there is no evidence of coronary inflammation in our cohort. However, coronary inflammation plays a relevant role both in myocarditis and atherosclerosis, but through different mechanisms; this may lead to different pFAI reference values according to different underlying diseases and might explain the lower pFAI values found in our cohort of patients with myocarditis.

As validated by histology, coronary inflammation in atherosclerosis follows an “outside to inside” model [6,7,8], with pro-inflammatory cytokines propagating towards the vessel’s lumen, inhibiting pre-adipocytes differentiation, and promoting lipolysis and oedema. Therefore, inflamed pericoronary adipose tissue is enriched in smaller adipocytes, located closer to the vascular wall, where the ratio of water/lipid is higher; this explains why, in inflamed coronaries, there is a shift towards higher pFAI values (towards −30 HU) [5,6,7,8]. Vascular inflammation in myocarditis, although still poorly explored, is thought to act both at the microcirculatory and at the epicardial level. Some viruses (i.e., parvovirus B19, PVB19) exert a direct pathogenic role on endothelial cells, and endothelial expression of adhesion molecules and human leukocyte antigens (HLA) is increased by pro-inflammatory cytokines [21,22,23,24]. The effect of pro-inflammatory cytokines on coronary endothelium determines increased vascular permeability (oedema) and a pro-coagulant environment, through the increased expression of tissue factor and endothelial cells necrosis, leading to vascular thrombosis.

The lower mean RCA pFAI values in our cohort may be explained by the different mechanisms of vascular inflammation, which is mainly endothelial in myocarditis. Although, by definition, there is no evidence of atherosclerotic coronary lesions in clinically suspected and biopsy-proven myocarditis, microcirculatory and epicardial inflammation may determine vasospasm and micro-thrombosis, which can explain chest pain and electrocardiographic changes in myocarditis. Coronary spasm and inflammation-mediated endothelial dysfunction have been proven in PVB19 myocarditis.

Only three patients later underwent an endomyocardial biopsy (EMB) in our cohort, of whom one had PVB19, while the remaining two were virus-negative. This suggests that endothelial inflammation in clinically suspected myocarditis might also be mediated by the immune response, as shown by the increased expression of adhesion molecules and HLA on EMB [21].

Of note, in our cohort, patients with higher pFAI values had greater ESV and lower RVEF on CMR (Table 3), as compared to those with lower pFAI values, suggesting that higher pFAI values in myocarditis may also identify patients with worse clinical features (larger ventricular volumes and lower systolic function). The cut-off for normal pFAI values in myocarditis may be different than that in atherosclerosis; if confirmed in larger studies, pFAI values may be of prognostic values in myocarditis as in CAD.

### Limitations

The main limitations of our study are the retrospective design and the small sample size that require any conclusion to be drawn cautiously. However, this study could serve as a pilot study to further confirm and expand our findings in a larger population.

Only three patients later underwent an EMB in our cohort; it will be interesting to explore pFAI values in a larger population of EMB-proven myocarditis patients, to confirm or exclude correlations between pFAI and specific endotheliotropic viruses. Moreover, histology data may help understand the link, if any exists, between local and systemic inflammation, which is still a debated issue.

## 5. Conclusions

In clinically suspected myocarditis with infarct-like presentation mean pFAI values, not different from those of healthy controls, would exclude the presence of coronary inflammation, based on pFAI values in coronary atherosclerosis, but a different pFAI cut-off value may be needed in these patients. The correlation between higher RCA mean pFAI values, higher biventricular ESV, and lower RVEF by CMR might suggest a potential role of pFAI in predicting non-atherosclerotic coronary inflammation, which is an infectious or immune-mediated “endothelialitis”, in patients with clinically suspected myocarditis and infarct-like presentation.

## Figures and Tables

**Table 1:** Clinical, demographic, laboratory, and imaging characteristics of patients with clinically suspected myocarditis undergoing and not undergoing CCTA.

	Clinically Suspected Myocarditis Undergoing CCTA *n* = 15	Clinically Suspected Myocarditis Not Undergoing CCTA *n* = 450	*p*-Value
Sex, female	5 (33)	127 (28)	0.67
Age at diagnosis, years	30 ± 10	36 ± 15	0.16
Family history of immune-mediated disease	3 (20)	63 (14) *	0.53
Acute viral infection 6 months before	3 (21) **	225 (51) *	0.032
Immune-mediated diseases	0 (0)	51 (11)	0.17
Diabetes	0 (0)	6 (1)	0.65
Clinical presentation			
Arrhythmias	1 (7) #	28 (6)	0.94
Heart failure	1 (7) #	29 (6)	0.97
Infarct-like	13 (87)	380 (84)	0.81
Asymptomatic	0 (0)	10 (2)	0.56
Symptoms			
Palpitations	2 (13)	31 (7) ***	0.35
Syncope	3 (20)	16 (4) ***	0.002
Chest pain	9 (64) **	250 (56) ^	0.56
Embolism	1 (7)	1 (0.2) ***	<0.001
NYHA class			0.031
I	12 (80)	419 (94) ^^	
II-IV	3 (20) ##	28 (6)	
Peak hs-Troponin I level, ng/L	5197 (364–8469)	331 (88–1181)	0.12
Peak C reactive protein level, mg/dL	16.4 (6.1–40.0) **	25.6 (7.5–63.8) °	0.27
AHA positivity	4/10 (40)	184/374 (49)	0.57
LA antero-posterior diameter, mm	50.8 ± 8.5 ^^^	41.1 ± 11.0 °°	0.007
LVEDV Echo, mL/m^2^	65 ± 13 °°°	63 ± 16 §	0.43
LVEF, %	56.4 ± 9.5 **	56.2 ± 9.5 §§	0.80
RVEDA, cm^2^	22.2 ± 3.4 §§§	20.3 ± 4.1 ¶	0.097
FAC, %	42.6 ± 7.8 §§§	45.1 ± 7.6 ¶¶	0.68
LVEDV CMR, mL/m^2^	86 ± 13 °°°	86 ± 18 ¶¶¶	0.96
LVESV CMR, mL/m^2^	36.8 ± 6.6 °°°	37.0 ± 12.0 ¶¶¶	0.74
RVEDV, mL/m^2^	87 ± 13 °°°	81 ± 17 ¶¶¶	0.16
RVESV, mL/m^2^	38.8 ± 8.4 °°°	33.2 ± 9.7 ¶¶¶	0.058
LVEF CMR, %	57.3 ± 4.0 °°°	57.6 ± 6.6 ¶¶¶	0.49
RVEF CMR, %	56.0 ± 4.7 °°°	60.0 ± 6.8 ¶¶¶¶	0.048
Myocardial oedema	9 (60)	225/357 (63)	0.47
Myocardial LGE	12 (86) **	319/347 (92)	0.41

Data are expressed as *n* (%), mean ± SD or median (IQR) as appropriate. NYHA, New York Heart Association; AHA, anti-heart antibody; LA, left atrium; LVEDV, left ventricular end-diastolic volume; LVEF, left ventricular ejection fraction; RVEDA, right ventricular end-diastolic area; FAC, fractional area change; LVESV, left ventricular end-systolic volume; RVEDV, right ventricular end-diastolic volume; RVESV, right ventricular end-systolic volume; LGE, late gadolinium enhancement. # In addition to infarct-like presentation (i.e., high Troponin and normal coronary arteries plus heart failure in 1 case and plus arrhythmia in 1 case). ## Only 1 patient fulfilled the 2016 ESC heart failure definition. * data available in 445 patients, ** data available in 14 patients, *** data are available in 442 patients, ^ data available in 443 patients, ^^ data available in 447 patients, ^^^ data available in 12 patients, ° data available in 258 patients, °° data available in 221 patients; °°° data available in 13 patients; § data available in 319 patients; §§ data available in 389 patients; §§§ data available in 10 patients; ¶ data available in 143 patients; ¶¶ data available in 127 patients; ¶¶¶ data available in 87 patients; ¶¶¶¶ data available in 85 patients.

**Table 2:** Mean pFAI values and vessel diameters of the three major coronary arteries.

	Observer 1		Observer 2		ICC pFAI Value			ICC Vessel Diameter		
	pFAI Value (HU)	Vessel Diameter (mm)	pFAI Value (HU)	Vessel Diameter (mm)	ICC	95% CI	*p*-Value	ICC	95% CI	*p*-Value
RCA	−92.8 ± 8.4	3.38 ± 0.74	−92.9 ± 8.3	3.51 ± 0.97	0.994	0.981–0.998	<0.01	0.851	0.623–0.947	<0.01
LAD	−90.3 ± 6.1	4.15 ± 0.88	−91.1 ± 6.2	3.91 ± 0.86	0.945	0.839–0.982	<0.01	0.851	0.623–0.947	<0.01
LCx	−86.4 ± 9.0	3.74 ± 1.01	−86.1 ± 9.4	3.70 ± 0.59	0.958	0.780–0.993	<0.01	0.825	0.253–0.968	<0.01

Data are expressed as mean ± SD. ICC, intraclass correlation coefficient; RCA, right coronary artery; LAD, left anterior descending artery; LCx, left circumflex artery; pFAI, pericoronary fat attenuation index.

**Table 3:** Demographic, clinical, and imaging characteristics according to RCA median pFAI values.

	Group 1 pFAI Values < RCA Median pFAI Value *n* = 7	Group 2 pFAI Values ≥ RCA Median pFAI Value *n* = 8	*p*-Value
Sex, male	4 (57)	6 (75)	0.46
Age at diagnosis, years	32.7 ± 10.5	27.6 ± 9.9	0.34
Family history of immune-mediated disease	3 (43)	0 (0)	0.038
Acute viral infection 6 months before	2 (29)	1 (14)	0.52
Symptoms before diagnosis	5 (71)	4 (50)	0.4
Palpitations	1 (14)	1 (12)	0.92
Syncope	1 (14)	2 (25)	0.6
Chest pain	5 (71)	4 (57)	0.58
Embolism	1 (14)	0 (0)	0.3
NYHA class			0.63
I	6 (86)	6 (75)	
II-IV	1 (14)	2 (24)	
Peak hs-Troponin I level, ng/L	5884 (4438–10,554)	2442 (146–7078)	0.26
C reactive protein level at diagnosis, mg/dL	28.2 (8.7–59.0)	10.0 (4.0–35.2)	0.50
AHA positivity	1 (5) *	3 (60) *	0.20
LVEDV Echo, mL/m^2^	62 ± 15	68 ± 11	0.46
LVEF Echo, %	60.6 ± 6.4	52.3 ± 10.8	0.095
LVEDV CMR, mL/m^2^	82 ± 14	90 ± 12 **	0.18
LVESV CMR, mL/m^2^	33.5 ± 4.0	39.6 ± 7.3 **	0.038
LVEF CMR, %	58.5 ± 4.4	56.3 ± 3.7 **	0.32
RVEDV CMR, mL/m^2^	83 ± 13	91 ± 13 **	0.34
RVESV, mL/m^2^	32.8 ± 3.2	43.9 ± 8.2 **	0.024
RVEF CMR, %	58.8 ± 3.3	53.6 ± 4.4 **	0.038
LGE presence	6 (86)	6 (75) **	0.38
Pleural effusion	1 (17)	1 (14) **	0.91

Data are expressed as *n* (%), mean ± SD or median (IQR), as appropriate. NYHA, New York Heart Association; AHA, anti-heart antibody; LVEDV, left ventricular end-diastolic volume; LVEF, left ventricular ejection fraction; LVESV, left ventricular end-systolic volume; RVEDV, right ventricular end-diastolic volume; RVESV, right ventricular end-systolic volume; RVEF, right ventricular ejection fraction. * data available in 5 patients, ** data available in 7 patients.

## Data Availability

Data available upon reasonable request to the corresponding author.
